# Supplementary material for: Novel Crosslinking System for Poly-Chloroprene Rubber to Enable Recyclability and Introduce Self-Healing
Source: Polymers (Basel). 2021 Sep 29;13(19):3347. doi: 10.3390/polym13193347 (PMC8512348; doi:10.3390/polym13193347)
Supplement: Supplementary file 1 [file polymers-13-03347-s001.zip › polymers-1392812-supplementary.pdf]

Supplementary Material

Novel Crosslinking System for Poly-Chloroprene Rubber to Enable Recyclability and Introduce Self-Healing

Anureet Kaur [1], Julien E. Gautrot [1], Gabriele Cavalli [1], Douglas Watson [2], Alan Bickley [2], Keizo Akutagawa [1] and James J. C. Busfield [1,*]

[1] School of Engineering and Materials Science, Queen Mary University of London, E1 4NS London, UK; Anureet.kaur@qmul.ac.uk (A.K.); j.gautrot@qmul.ac.uk (J.E.G.); g.cavalli@qmul.ac.uk (G.C.); akutag@jcom.home.ne.jp (K.A.)

[2] Weir Advanced Research Centre, G1 1RD Glasgow, UK; douglas.watson@mail.weir (D.W.); alan.bickley@mail.weir (A.B.)

* Correspondence: j.busfield@qmul.ac.uk

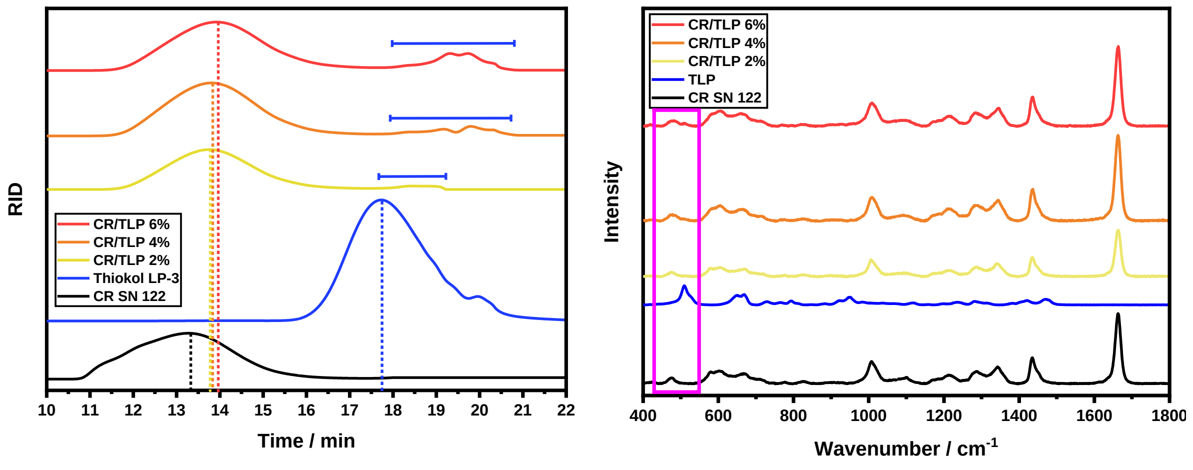

(**a**) (**b**)

Figure S1. Formulations analysis comparison before curing between CR, Thiokol LP-3 and all compounds after mixing on the two roll mill: **(a)** GPC analysis; **(b)** Raman spectroscopy.

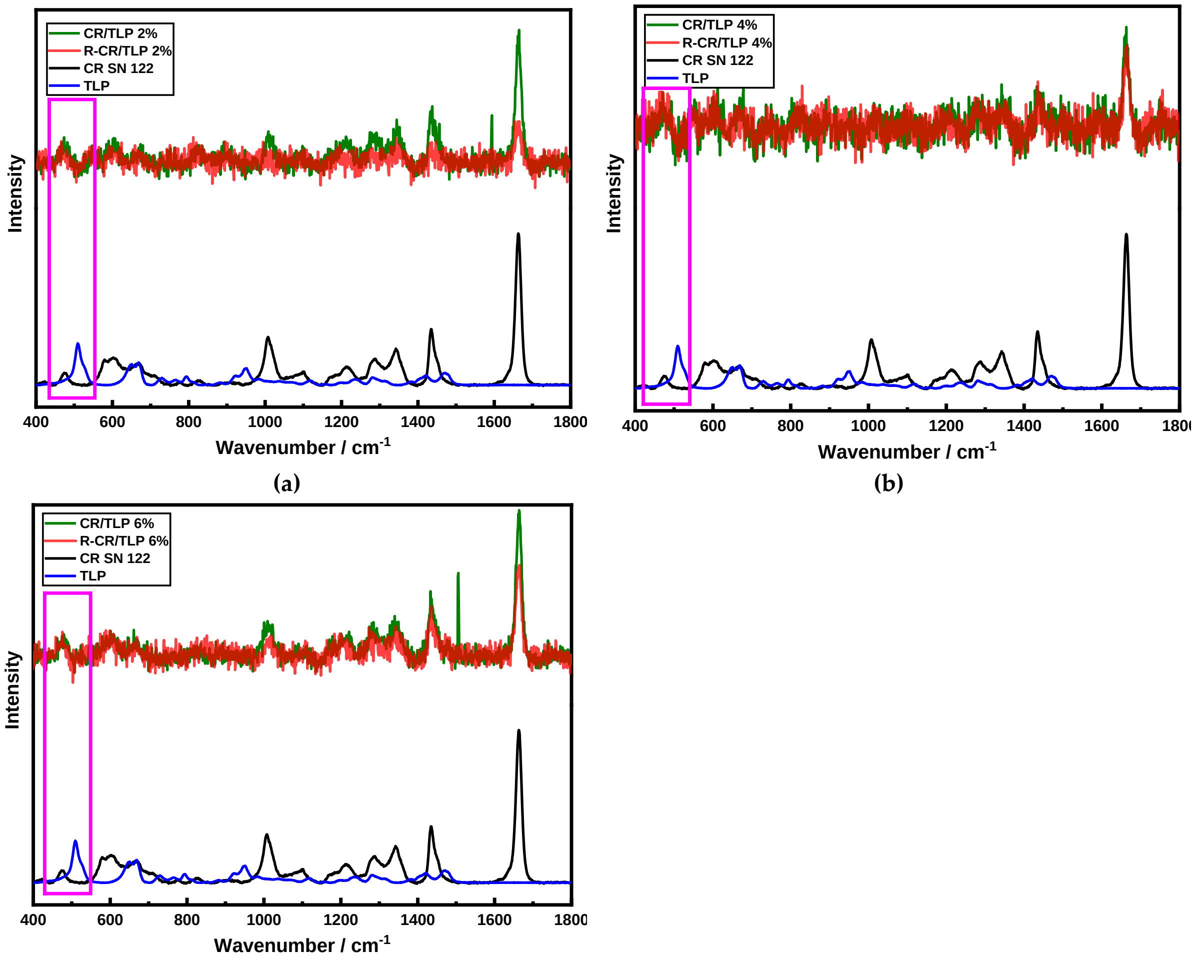

(**a**) (**b**)

(**c**)

Figure S2. Raman analysis on cured compounds

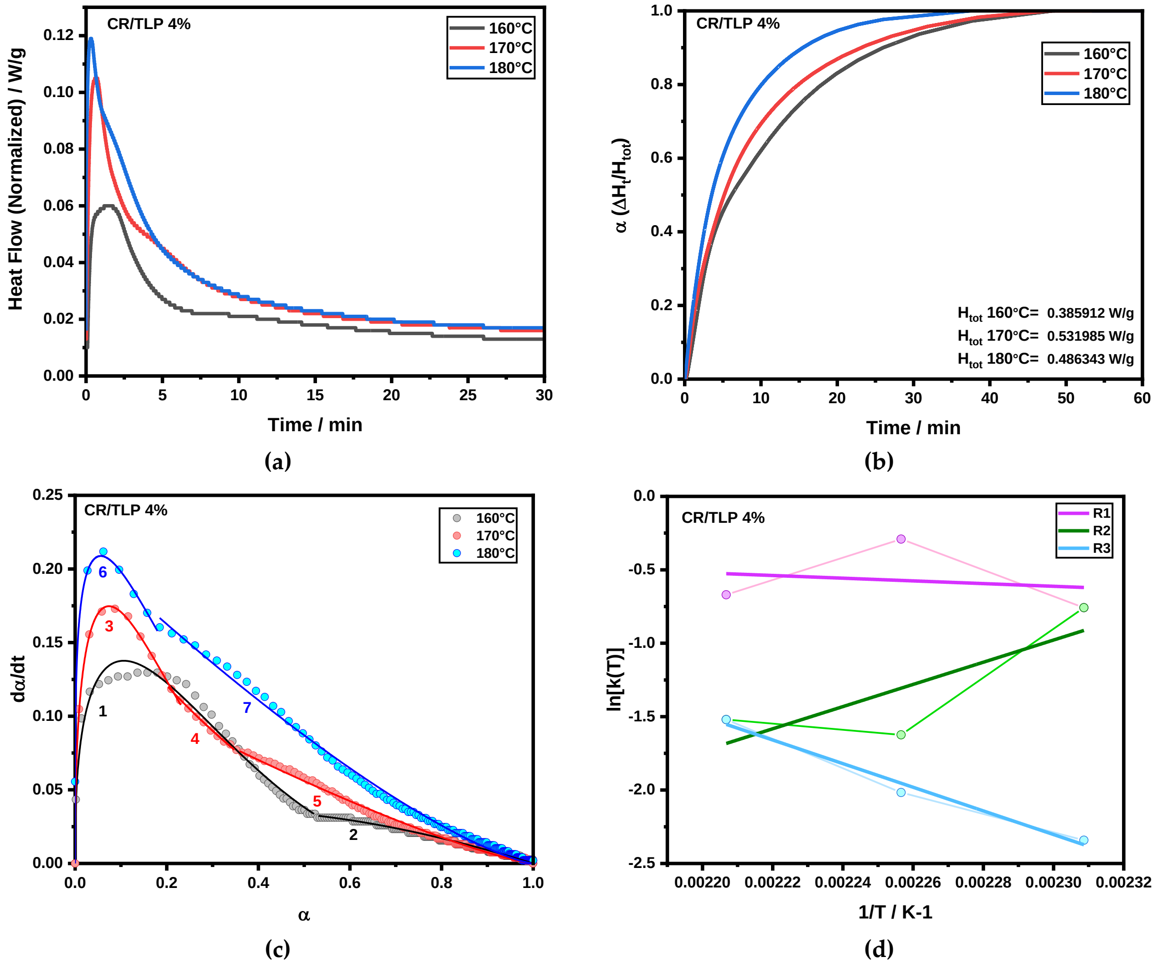

Figure S3. Kinetics and curing parameters derived from isothermal DSC analysis of compound CR/TLP 4%: (**a**) heat flow vs. time at temperatures of 160°C, 170°C and 180°C; (**b**) degree of cure calculated; (**c**) autocatalytic model fitting for the domains of Reaction 1 (R1), Reaction 2 (R2) and Reaction 3 (R3); the indices from 1 to 7 are related to the fitting parameters reported in Table S1; (**d**) activation energy, E_a, and pre-exponential factor k_0 obtained from the slope and intercept values, respectively, of the linear regression for three distinctive reactions occurring during curing.

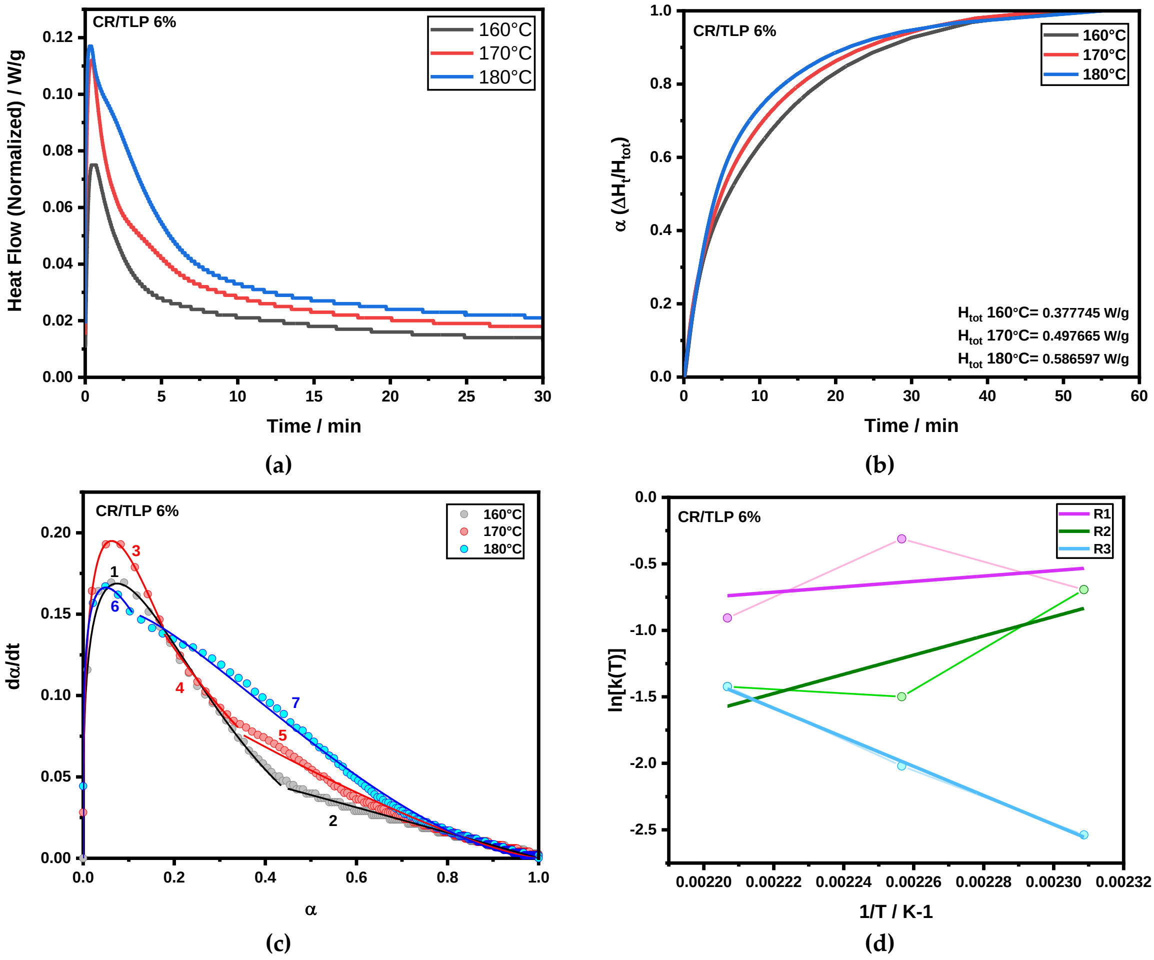

Figure S4. Kinetics and curing parameters derived from isothermal DSC analysis of compound CR/TLP 6%: (**a**) heat flow vs. time at temperatures of 160°C, 170°C and 180°C; (**b**) degree of cure calculated; (**c**) autocatalytic model fitting for the domains of Reaction 1 (R1), Reaction 2 (R2) and Reaction 3 (R3); the indices from 1 to 7 are related to the fitting parameters reported in Table S1; (**d**) activation energy, E_a, and pre-exponential factor k_0 obtained from the slope and intercept values, respectively, of the linear regression for three distinctive reactions occurring during curing.

Table S1: Kinetic parameters obtained from isothermal DSC analysis of CR/TLP 2%, CR/TLP 4% and CR/TLP 6%

	T /°C		***k(T)***		***m***		***n***		χ_r^2	R^2	R_{adj}^2
			value	±	***value***	±	***value***	±			
**CR/TLP 2%**	**160**	**1**	0.2652	0.00346	0.26446	0.00304	3.16023	0.06094	1.33411E-5	0.98192	0.2652
		**2**	1.45296	0.03246	1	0.0104	4.3136	0.02607	7.66952E-6	0.98522	1.45296
		**3**	0.09077	1.03172E-4	0	0	1.00096	9.16612E-4	1.37102E-6	0.98993	0.09077
	**170**	**4**	0.56903	0.00374	0.39776	0.00164	4.2702	0.02576	4.34717E-6	0.99671	0.56903
		**5**	0.17658	2.1224E-4	0	0	1.98083	0.004	6.377E-7	0.99584	0.17658
		**6**	0.13894	1.1465E-4	0	0	1.2172	8.21707E-4	1.73651E-6	0.99377	0.13894
	**180**	**7**	0.47441	0.00437	0.26404	0.0022	4.73394	0.03922	1.15581E-5	0.97139	0.47441
		**8**	0.18791	1.21781E-4	0	0	1.49318	9.25663E-4	3.66522E-6	0.99402	0.18791
**CR/TLP 4%**	**160**	**1**	0.46879	0.00305	0.38452	0.0022	3.23446	0.01044	2.86043E-5	0.97904	0.46879
		**2**	0.09616	5.63667E-4	0.52493	0.00759	1	0.00242	1.02926E-6	0.98803	0.09616
	**170**	**3**	0.74747	0.00273	0.40736	9.72413E-4	5.11139	0.01138	2.83669E-6	0.99788	0.74747
		**4**	0.19713	2.9705E-4	0	0	2.19419	0.0047	6.0821E-7	0.99592	0.19713
		**5**	0.13312	1.38027E-4	0	0	1.25032	0.00106	2.54111E-6	0.99015	2
	**180**	**6**	0.51159	0.00505	0.23251	0.00241	3.92074	0.03853	1.99494E-5	0.95967	0.51159
		**7**	0.21865	1.76945E-4	0	0	1.32883	0.00107	6.82034E-6	0.99403	0.21865
**CR/TLP 6%**	**160**	**1**	0.49992	0.00197	0.305	0.0012	3.79238	0.00791	1.09025E-5	0.99356	0.49992
		**2**	0.07903	4.1902E-4	0.02246	0.00578	1	0.00242	1.35221E-6	0.9884	0.07903
	**170**	**3**	0.73215	0.0048	0.35353	0.00162	5.31346	0.02557	6.67565E-6	0.99241	0.73215
		**4**	0.22336	4.31667E-4	0	0	2.46139	0.00649	1.67387E-6	0.99369	0.22336
		**5**	0.13263	1.70452E-4	0	0	1.29564	0.00135	3.71359E-6	0.98504	0.13263
	**180**	**6**	0.40386	0.00546	0.22287	0.00295	4.27359	0.0794	1.3403E-5	0.96186	0.40386
		**7**	0.24101	6.36345E-4	0.12663	0.00148	1.62614	0.00199	3.92051E-6	0.99468	0.24101

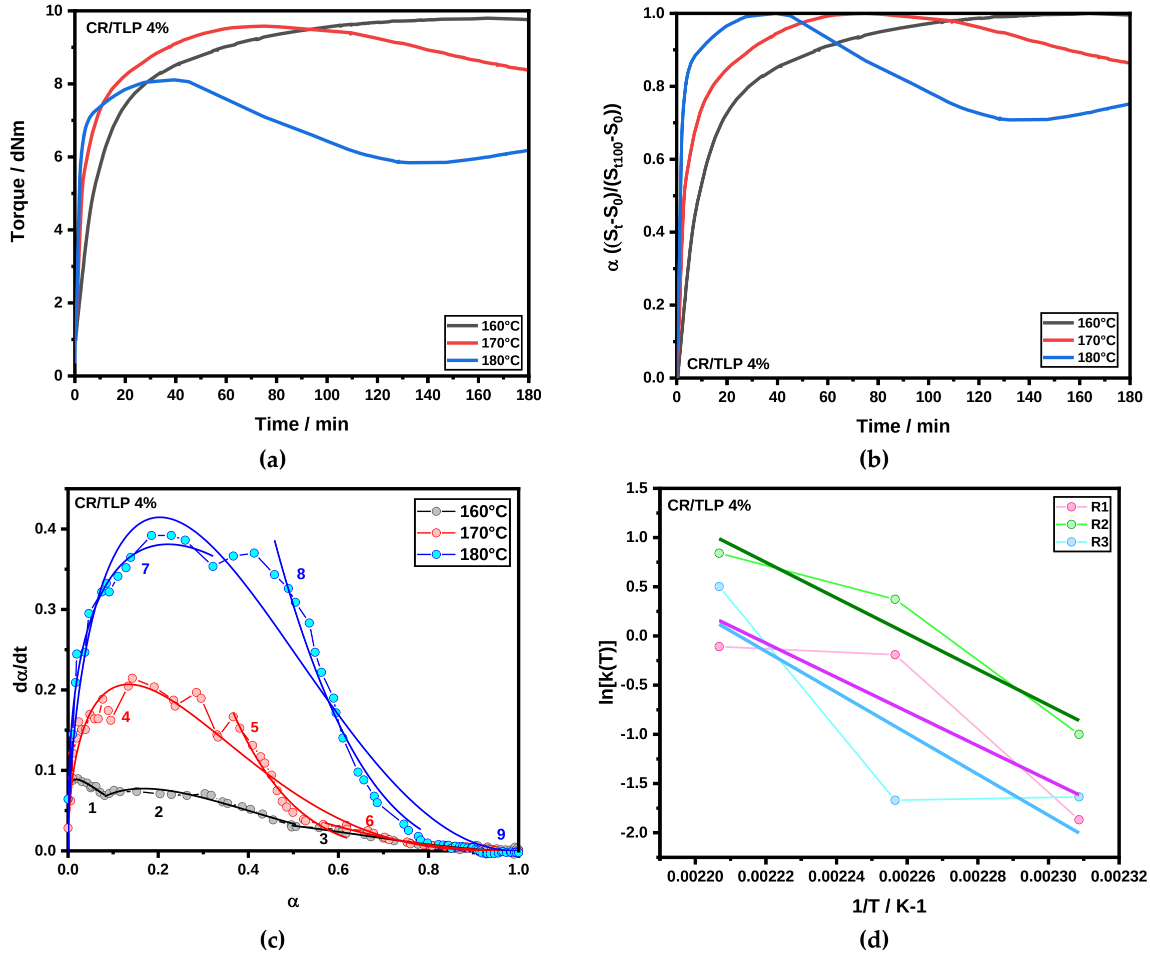

Figure S5. Kinetics and curing parameters derived from rheology of compound CR/TLP 4%: (**a**) Torque vs. time at temperatures of 160°C, 170°C and 180°C; (**b**) degree of cure calculated; (**c**) autocatalytic model fitting for the domains of Reaction 1 (R1), Reaction 2 (R2) and Reaction 3 (R3); the indices from 1 to 9 are related to the fitting parameters reported in Table S2; (**d**) activation energy, E_a, and pre-exponential factor k_0 obtained from the slope and intercept values, respectively, of the linear regression for three distinctive reactions occurring during curing.

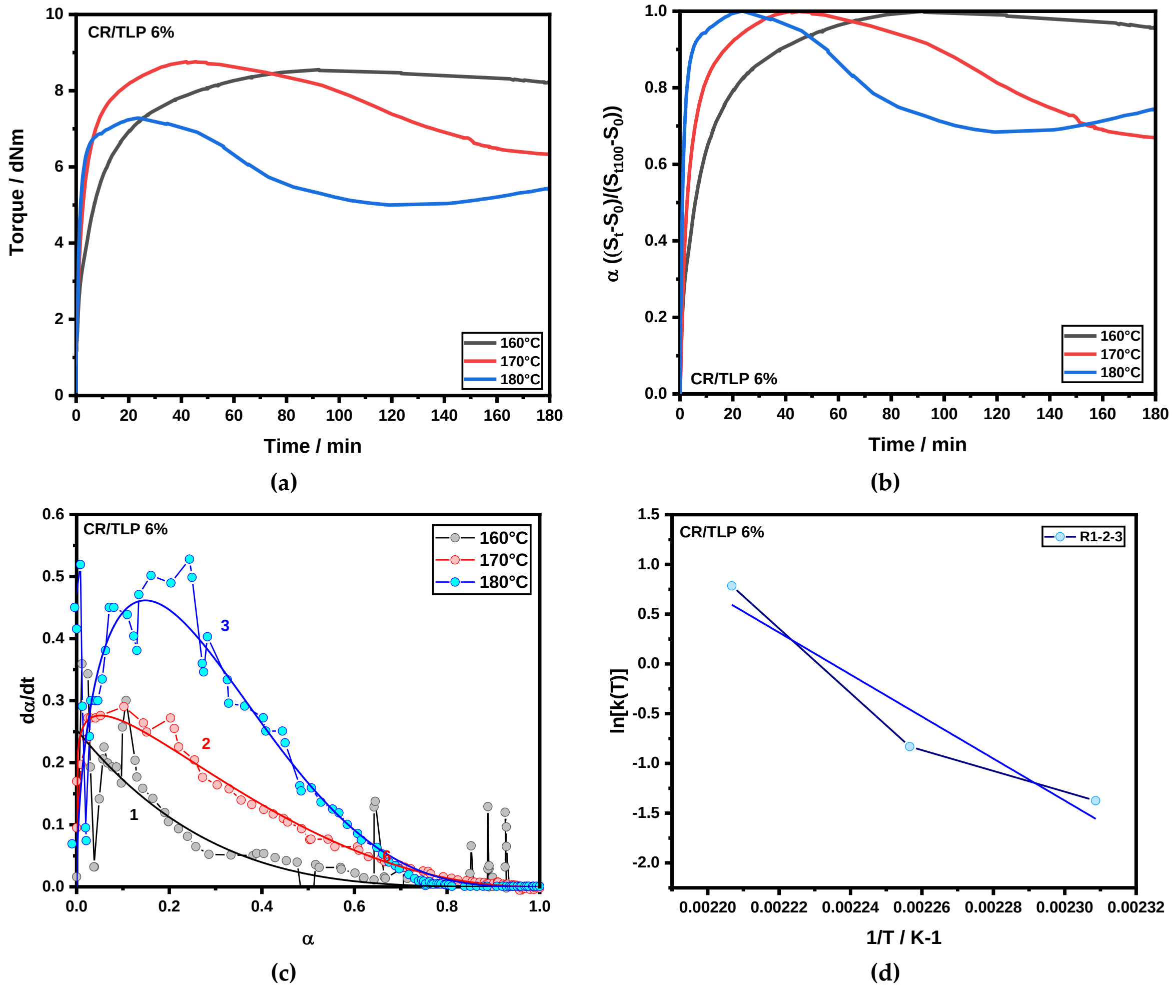

Figure S6. Kinetics and curing parameters derived from rheology of compound CR/TLP 6%: (**a**) Torque vs. time at temperatures of 160°C, 170°C and 180°C; (**b**) degree of cure calculated; (**c**) autocatalytic model fitting for one single domain where Reaction 1 (R1), Reaction 2 (R2) and Reaction 3 (R3) appears to be controlled by the same curing and kinetic parameters; the indices from 1 to 3 are related to the fitting parameters reported in Table S2; (**d**) activation energy, E_a, and pre-exponential factor k_0 obtained from the slope and intercept values, respectively, of the linear regression for three distinctive reactions occurring during curing.

Table S2: Kinetic parameters obtained from curing-Rheology analysis of CR/TLP 2%, CR/TLP 4% and CR/TLP 6%

	T /°C		*k(T)*		*m*		*n*		$\chi_r^{\ 2}$	R^2	R_{adj}^2
			value	±	*value*	±	*value*	±			
**CR/TLP 2%**	**160**	**1**	0.46593	0.17693	0.75751	0.12733	1	0.75465	5.85626E-5	0.95032	0.46593
		**2**	1.62569	1.0885	0	0	7.44687	1.6434	3.05168E-4	0.75961	1.62569
		**3**	0.13134	0.07622	1	0.53648	1.57357	0.31534	4.38476E-6	0.9314	0.13134
	**170**	**4**	2.57014	0.59906	1	0.07318	2.58462	0.53546	1.02018E-4	0.98812	2.57014
		**5**	2.698	0.62142	0	0	8.04655	0.70048	5.66908E-4	0.88699	2.698
		**6**	0.12298	0.02347	0	0	1.55766	0.27609	1.90792E-4	0.703	0.12298
	**180**	**7**	1.72624	0.44918	0.68902	0.07867	1.6316	0.62181	5.27636E-4	0.96403	1.72624
		**8**	6.62253	1.43205	0	0	8.21458	0.55365	4.71623E-4	0.92969	6.62253
		**9**	0.20294	0.02242	0	0	2.02098	0.13675	3.28587E-5	0.90553	0.20294
**CR/TLP 4%**	**160**	**1**	0.15448	0.02761	0.10992	0.03964	6.17432	1.10917	3.30205E-6	0.92955	0.15448
		**2**	0.36807	0.06978	0.5785	0.07595	2.87093	0.24897	1.22346E-5	0.95214	0.36807
		**3**	0.19488	0.12432	0.77278	0.62853	1.87094	0.33528	4.76393E-6	0.94175	0.19488
	**170**	**4**	0.82599	0.08857	0.47172	0.0347	3.03367	0.16798	2.28691E-4	0.95866	0.82599
		**5**	1.45111	0.22818	0	0	4.67278	0.28702	7.86799E-5	0.96405	1.45111
		**6**	0.18837	0.03566	0	0	2.00072	0.17479	8.25093E-6	0.9128	0.18837
	**180**	**7**	0.89764	0.15557	0.35943	0.05226	1.25923	0.38783	5.65365E-4	0.93549	0.89764
		**8**	2.31942	0.32002	0	0	2.92661	0.18133	3.95844E-4	0.97045	2.31942
		**9**	1.65272	0.18082	0.5574	0.04235	2.17849	0.1224	9.38751E-4	0.95713	1.65272
**CR/TLP 6%**	**160**	**1**	0.25309	0.02831	0	0	3.63734	0.87675	0.00463	0.57544	0.25309
	**170**	**2**	0.43578	0.04218	0.11618	0.02767	2.12049	0.16836	6.58069E-4	0.92967	0.43578
	**180**	**3**	2.19264	0.59195	0.55047	0.09187	3.16304	0.41654	0.00746	0.76067	2.19264

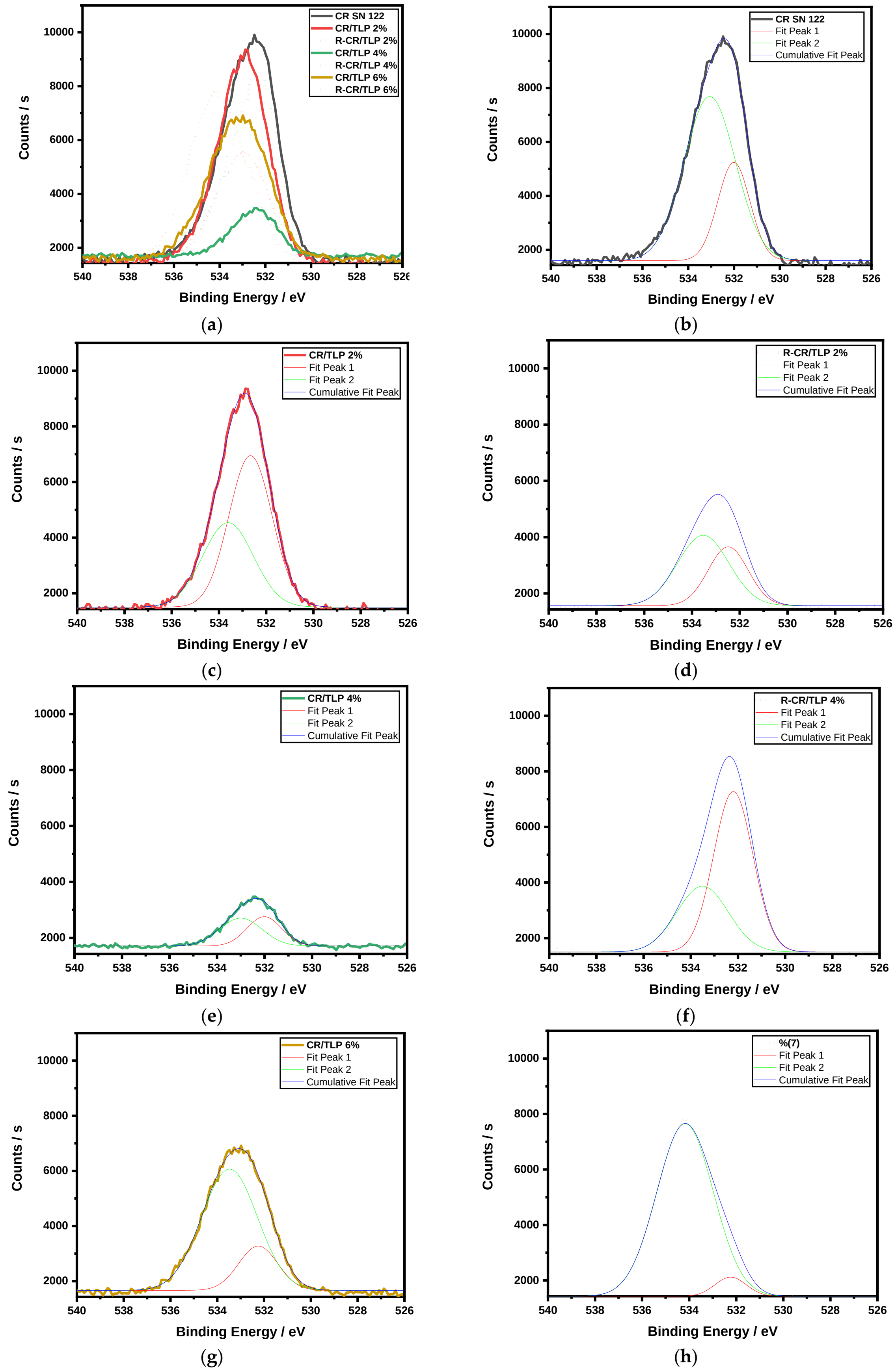

Figure S7: High resolution O 1s spectra of cured compounds and recycled compounds. (**a**) Comparison between all cured compounds, recycled compounds and CR SN 122; (**b**) (**c**) (**d**) (**e**) (**f**) (**g**) (**h**) deconvolution curves: green solid curves related to organic-oxygen and red solid curves are related to inorganic-oxygen

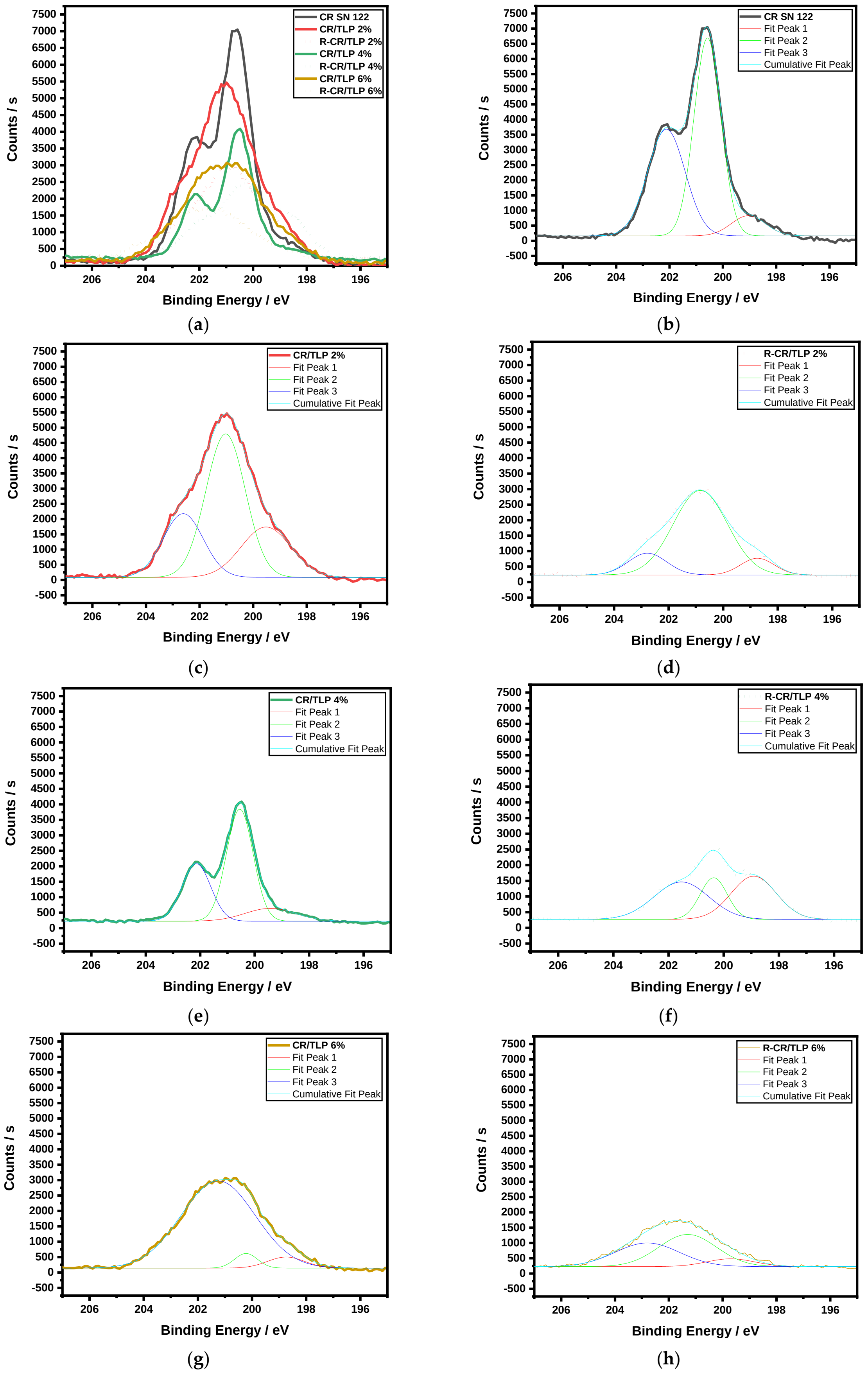

Figure S8: High resolution Cl 2p spectra of cured compounds and recycled compounds. (**a**) Comparison between all cured compounds, recycled compounds and CR SN 122; (**b**) (**c**) (**d**) (**e**) (**f**) (**g**) (**h**) deconvolution curves: blue and green solid curves related to organic-chlorine and red solid curves are related to inorganic-chlorine.

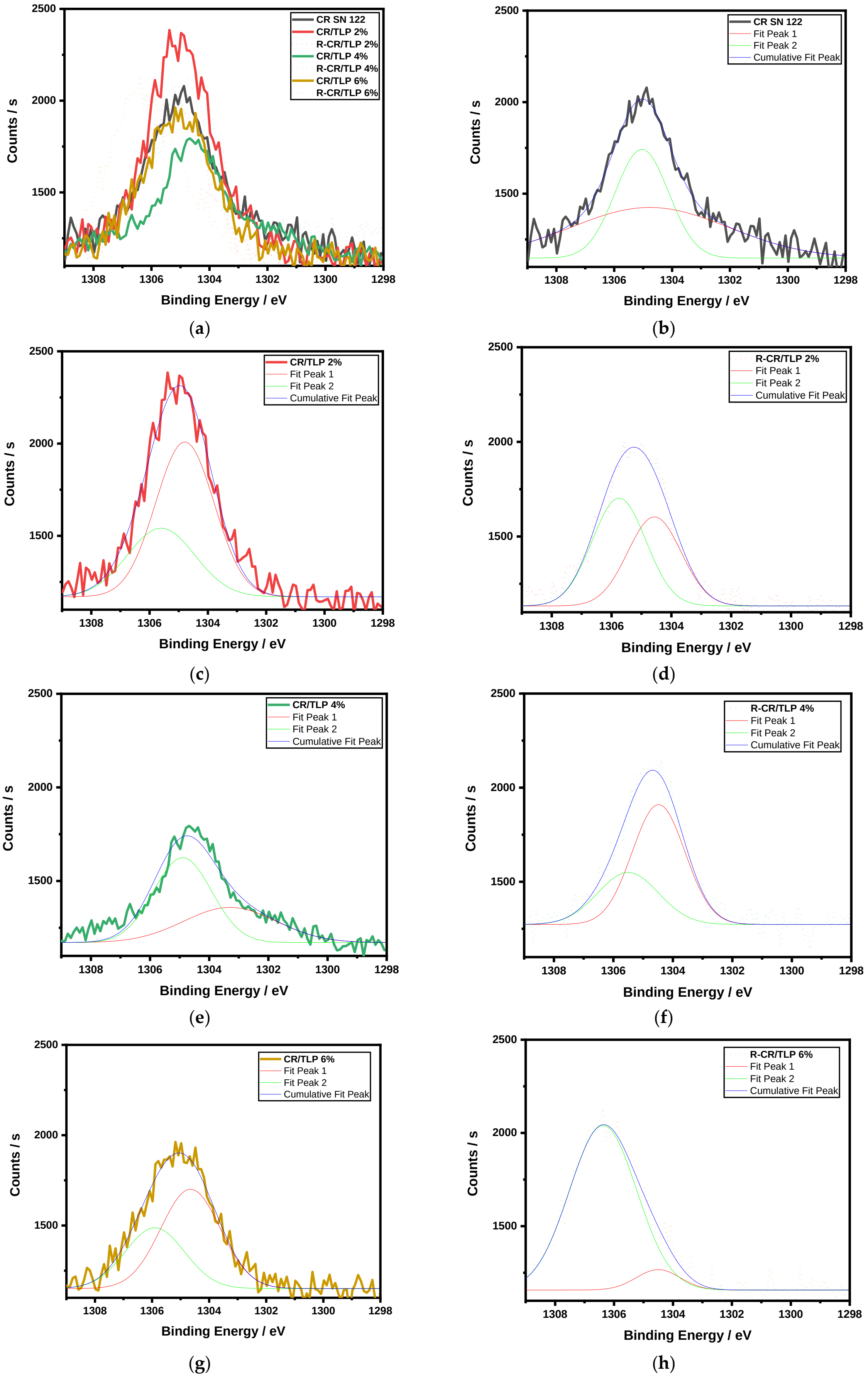

Figure S9: High resolution Mg 1s spectra of cured compounds and recycled compounds. (**a**) Comparison between all cured compounds, recycled compounds and CR SN 122; (**b**) (**c**) (**d**) (**e**) (**f**) (**g**) (**h**) deconvolution curves: green solid curves related to magnesium chloride and red solid curves are related to magnesium oxide.

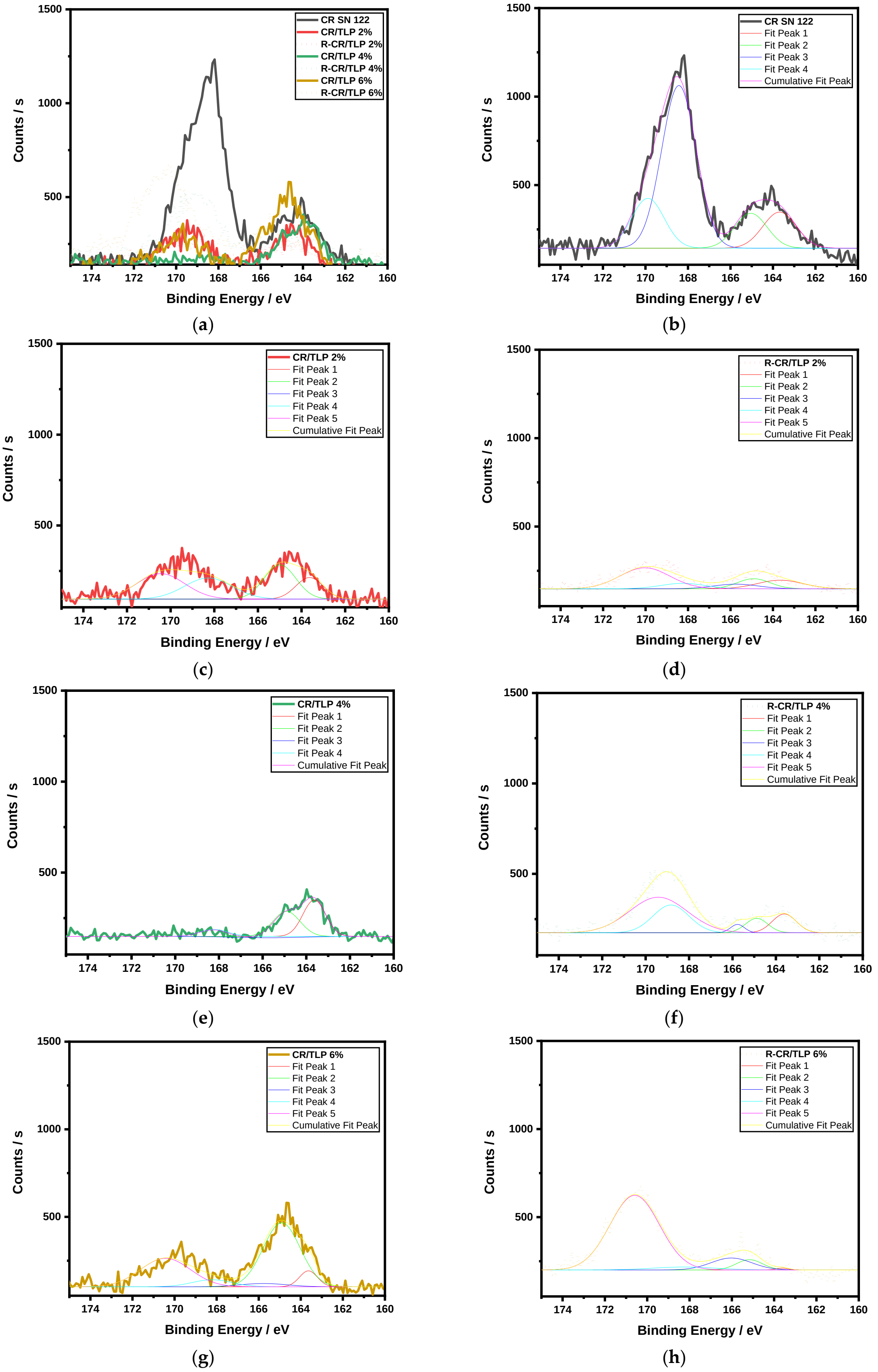

Figure S10: High resolution S 2p spectra of cured compounds and recycled compounds. (**a**) Comparison between all cured compounds, recycled compounds and CR SN 122; (**b**) (**c**) (**d**) (**e**) (**f**) (**g**) (**h**) deconvolution curves: blue, green and red solid curves related to organic-sulfur and light blue and pink solid curves are related to inorganic sulfur.

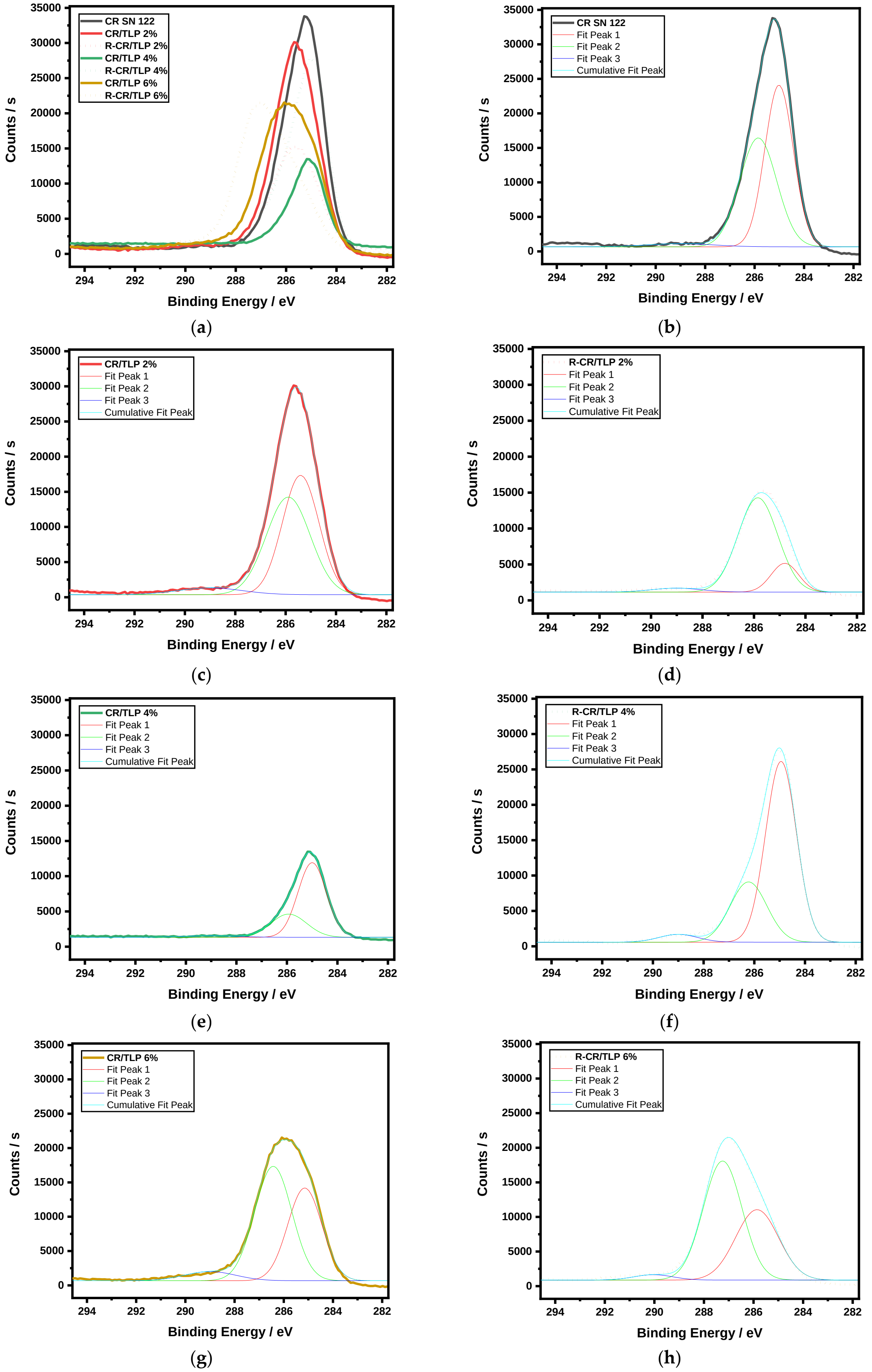

Figure S11: High resolution C 1s spectra of cured compounds and recycled compounds. (**a**) Comparison between all cured compounds, recycled compounds and CR SN 122; (**b**) (**c**) (**d**) (**e**) (**f**) (**g**) (**h**) deconvolution curves: blue solid curves related to carbon bonded to chlorine atoms, green solid curves are related to carbon atoms bonded to oxygen and red solid curves are related to the organic structure.

Table S3: O 1s spectra analysis: calculation of area under each deconvoluted peak.

	**Inorganic-Oxide**		**Organic-O**			**Inorganic-Oxide**		**Organic-O**	
	**peak / eV**	**area / %**	**peak / eV**	**area /%**		**peak / eV**	**area / %**	**peak / eV**	**area / %**
CR SN 122	532	**27%**	533	**73%**					
CR/TLP 2%	532.7	**60%**	532.6	**40%**	R-CR/TLP 2%	532.5	**39%**	533.5	**61%**
CR/TLP 4%	532	**54%**	533	**46%**	R-CR/TLP 4%	532.5	**65%**	533.5	**35%**
CR/TLP 6%	532.3	**20%**	533.5	**80%**	R-CR/TLP 6%	532.2	**6%**	534.2	**94%**

Table S4: Cl 2p spectra analysis: calculation of area under each deconvoluted peak.

	**Inorganic**		**Organic**		**Organic**	
	**peak / eV**	**area / %**	**peak / eV**	**area /%**	**peak / eV**	**area /%**
CR SN 122	199	**7%**	200.6	**53%**	202.1	**40%**
CR/TLP 2%	199.5	**23%**	201	**53%**	202.6	**24%**
CR/TLP 4%	199.4	**12%**	200.5	**56%**	202.1	**31%**
CR/TLP 6%	198.7	**6%**	200.2	**5%**	201.2	**90%**
	**peak / eV**	**area / %**	**peak / eV**	**area / %**	**peak / eV**	**area / %**
R-CR/TLP 2%	198.7	**9%**	200.8	**77%**	202.8	**14%**
R-CR/TLP 4%	198.9	**38%**	200.4	**22%**	201.5	**40%**
R-CR/TLP 6%	199.7	**10%**	201.3	**49%**	202.8	**41%**

Table S5: Mg 1s spectra analysis: calculation of area under each deconvoluted peak.

	**Mg-Oxide**		**Mg-Chloride**			**Mg-Oxide**		**Mg-Chloride**	
	**peak / eV**	**area / %**	**peak / eV**	**area /%**		**peak / eV**	**area / %**	**peak / eV**	**area / %**
CR SN 122	1304.8	**48%**	1305	**52%**					
CR/TLP 2%	1304.8	**67%**	1305.6	**33%**	R-CR/TLP 2%	1304.6	**45%**	1305.7	**55%**
CR/TLP 4%	1303.3	**40%**	1304.9	**60%**	R-CR/TLP 4%	1304.5	**67%**	1305.5	**33%**
CR/TLP 6%	1304.7	**63%**	1305.9	**37%**	R-CR/TLP 6%	1304.5	**9%**	1306.8	**91%**

Table S6: S 2p spectra analysis: calculation of area under each deconvoluted peak.

	**deconvolution values Sulfur**									
	**C-Sx-C**		**C-S-S-C**		**C-S-H**		**Sulfite SO_3^{2-}**		**Sulfate SO_4^{2-}**	
	peak / eV	area / %	peak / eV	area /%	peak / eV	area /%	peak / eV	area /%	peak / eV	area /%
CR SN 122	163.7	**13%**	165	**12%**		**0%**	168.4	**59%**	169.9	**17%**
CR/TLP 2%	163.6	**15%**	165	**28%**		**0%**	168.3	**26%**	170.5	**31%**
CR/TLP 4%	163.6	**46%**	164.9	**43%**		**0%**	168.2	**11%**		**0%**
CR/TLP 6%	163.6	**6%**	164.9	**52%**	165.8	**3%**	168.2	**6%**	170.4	**31%**
	**peak / eV**	**area / %**	**peak / eV**	**area / %**	**peak / eV**	**area / %**	**peak / eV**	**area /%**	**peak / eV**	**area /%**
R-CR/TLP 2%	163.7	**18%**	164.9	**15%**	165.7	**9%**	168.2	**11%**	170.0	**47%**
R-CR/TLP 4%	163.6	**11%**	164.9	**8%**	165.8	**3%**	168.8	**25%**	169.4	**52%**
R-CR/TLP 6%	163.6	**0.5%**	165.2	**6%**	166	**10%**	168.3	**4%**	170.6	**79%**

Table S7: C 1s spectra analysis: calculation of area under each deconvoluted peak.

	**C-C-C**		**C-C-O**		**C-C-Cl**	
	**peak / eV**	**area / %**	**peak / eV**	**area /%**	**peak / eV**	**area /%**
CR SN 122	285	**52%**	285.9	**46%**	289	**2%**
CR/TLP 2%	285.4	**48%**	285.9	**47%**	289	**5%**
CR/TLP 4%	285	**70%**	285.9	**28%**	289	**3%**
CR/TLP 6%	285.1	**40%**	286.4	**54%**	289	**6%**
	**peak / eV**	**area / %**	**peak / eV**	**area / %**	**peak / eV**	**area / %**
R-CR/TLP 2%	284.8	**17%**	285.9	**79%**	289	**4%**

R-CR/TLP 4%	284.9	**69%**	286.2	**27%**	289	**4%**
R-CR/TLP 6%	285.9	**39%**	287.2	**59%**	290	**3%**
